# Alternative Strategy to Reduce Surface Recombination for InGaN/GaN Micro-light-Emitting Diodes—Thinning the Quantum Barriers to Manage the Current Spreading

**DOI:** 10.1186/s11671-020-03372-3

**Published:** 2020-08-06

**Authors:** Le Chang, Yen-Wei Yeh, Sheng Hang, Kangkai Tian, Jianquan Kou, Wengang Bi, Yonghui Zhang, Zi-Hui Zhang, Zhaojun Liu, Hao-Chung Kuo

**Affiliations:** 1 School of Electronics and Information Engineering, Key Laboratory of Electronic Materials and Devices of Tianjin, Hebei University of Technology, 5340 Xiping Road, Beichen District, Tianjin, 300401 China; 2 Department of Photonics and Institute of Electro-optical Engineering, National Chiao Tung University, Hsinchu, 30010 Taiwan; 3 Department of Electronic and Electrical Engineering, Southern University of Science and Technology, Shenzhen, 518055 China

**Keywords:** Micro-LED, Sidewall defects, Nonradiative recombination, Current spreading, Hole injection, IQE

## Abstract

Owing to high surface-to-volume ratio, InGaN-based micro-light-emitting diodes (μLEDs) strongly suffer from surface recombination that is induced by sidewall defects. Moreover, as the chip size decreases, the current spreading will be correspondingly enhanced, which therefore further limits the carrier injection and the external quantum efficiency (EQE). In this work, we suggest reducing the nonradiative recombination rate at sidewall defects by managing the current spreading effect. For that purpose, we properly reduce the vertical resistivity by decreasing the quantum barrier thickness so that the current is less horizontally spreaded to sidewall defects. As a result, much fewer carriers are consumed in the way of surface nonradiative recombination. Our calculated results demonstrate that the suppressed surface nonradiative recombination can better favor the hole injection efficiency. We also fabricate the μLEDs that are grown on Si substrates, and the measured results are consistent with the numerical calculations, such that the EQE for the proposed μLEDs with properly thin quantum barriers can be enhanced, thanks to the less current spreading effect and the decreased surface nonradiative recombination.

## Introduction

Owing to the distinctive characteristics of high brightness, low power consumption, and long operation lifetime [1], III-nitride-based light-emitting diodes (LEDs) have gained extensive research interest [2, 3]. Thus far, tremendous progress for large-size InGaN/GaN blue LEDs has been made and commercialized [3], which have found applications in solid-state lighting and large-size panel displays. However, conventional InGaN/GaN LEDs are of small modulation bandwidth, making them not proper for, e.g., visible light communication (VLC) [4–6]. Meanwhile, the large chip size makes the pixel capacity low for, e.g., cell phone displays, wearable watch displays. Therefore, at the current stage, InGaN/GaN micro-LEDs (i.e., μLEDs) with chip size smaller than 100 μm have attracted extensive attentions. Despite the aforementioned advantages, there are still many issues remaining to be solved for the further development of μLEDs, such as high-precision mass transfer [7–9] and size-dependent efficiency [10]. The size-dependent efficiency arises from surface damages that are caused by dry etching when making mesas, and hence large numbers of defects are generated, giving rise to nonradiative surface recombination. Note, for different types of optoelectronic devices, crystalline quality and charge transport are among the essential parameters than affect the photoelectronic properties [11–16]. Uniquely for μLEDs, the surface recombination at defected regions can significantly reduce the internal quantum efficiency (IQE) for μLEDs [17]. Recently, Kou et al. further find that as the chip size decreases, holes are more easily trapped by the defects and the hole injection capability can become even worse for μLEDs with decreasing chip size [18]. Thus, it is important to reduce the sidewall defect density. A very convenient method to passivate sidewall defects is to deposit the dielectric passivation layer [19], which is doable by using plasma-enhanced chemical vapor deposition (PECVD) method or atomic layer deposition (ALD) method. It is shown that the dielectric passivation layer can better annihilate sidewall defects by using ALD technique because of the even better quality for the grown layer [20]. The sidewall defect number can be then further decreased by thermally annealing the passivation layer [21], which shows the enhanced EQE even for the 6 μm × 6 μm μLED. As is well known, the current spreading can become even better when the chip size continues to decrease because of the reduced lateral resistivity [22]. Therefore, we propose to reduce the vertical resistivity to better confine the current within mesas, which then keeps the carriers apart from sidewall defects and helps to suppress the surface nonradiative recombination.

Hence, for achieving the target, we propose decreasing the thickness of quantum barriers to manage the energy barriers and the vertical resistance. Our numerical calculations show that the current can be more laterally confined into the mesa, which therefore reduces the hole consumption by surface nonradiative recombination. The reduced surface nonradiative recombination also helps to facilitate the hole injection according to our previous report [18]. Furthermore, the thinned quantum barriers homogenize the hole distribution across the multiple quantum wells (MQWs). Experimental results indicate that the EQE for μLEDs with reduced quantum barrier thickness is improved.

## Research Methods and Physics Models

To prove the effectiveness of the proposed structures in suppressing the surface recombination, promoting the hole injection and the improving the EQE for InGaN-μLEDs, different sets of μLEDs are designed, which are grown on [111] oriented Si substrates by using metal-organic chemical vapor deposition (MOCVD) system [23, 24]. All the devices have a 4-μm thick n-GaN layer with the electron concentration of 5 × 10^18^ cm^−3^. Then, four-pair In_0.18_Ga_0.82_N/GaN MQWs are utilized to produce photons. The structural information is presented in Table 1. Next, a 26-nm thick p-Al_0.15_Ga_0.85_N layer serves as the p-type electron blocking layer (p-EBL), for which the hole concentration level is 3 × 10^17^ cm^−3^, of the p-EBL is then capped with a 100-nm thick p-GaN layer with a hole concentration is 3 × 10^17^ cm^−3^. Finally, both μLED samples are covered by a 20-nm p-GaN layer. All the investigated InGaN-based blue μLEDs have the chip dimension of 10 × 10 μm^2^. The 200 nm ITO is utilized as the current spreading layer, which is annealed at the temperature of 500 °C for 120 s to form ohmic contact with p-GaN layer. Then Ti/Al/Ni/Au/ alloy is simultaneously deposited on the current spreading layer and the n-GaN layer serving as the p-electrode and the n-electrode, respectively.

**Table 1:** The structural parameters of the active region for μLEDs A, B, and C

MQWs	μLED A	μLED B	μLED C
GaN (QB)	6 nm	9 nm	12 nm
In_0.18_Ga_0.82_N(QW)	3 nm	3 nm	3 nm
GaN (QB)	6 nm	9 nm	12 nm

To reveal the device physics at an in-depth level, the investigated devices are calculated by using APSYS [25, 26], which can self-consistently solve drift-diffusion equations, Schrödinger and Poisson’s equations. The light extraction efficiency is set to 88.1% for flip-chip devices [27]. The energy band offset ratio between the conduction band and the valence band in the InGaN/GaN MQWs is set to 70:30 [28]. Carrier loss due to nonradiative recombination is also considered in our calculations, including Auger recombination with the recombination coefficient of 1 × 10^−30^ cm^6^s^−1^ and Shockley-Read-Hall (SRH) recombination with the carrier lifetime of 100 ns [29]. The nonradiative recombination occurring at mesa surfaces cannot be ignored for μLEDs. For accurately modeling the surface recombination, the trap levels for electrons and holes are set at 0.24 eV below the conduction band (i.e., E_c_ − 0.24 eV) and 0.46 eV above the valence band (i.e., E_v_ + 0.46 eV), respectively. The capture cross-section of 3.4 × 10^−17^ cm^2^ and the trap density of 1 × 10^13^ cm^−3^ are set for electron traps [30]. The capture cross-section of 2.1 × 10^−15^ cm^2^ and the trap density of 1.6 × 10^13^ cm^−3^ are set for holes [31]. Other parameters can be found elsewhere [32].

## Results and Discussions

### Proof of the Better Current Confinement Within the Mesa Region by Thinning Quantum Barriers for μLEDs

**Fig. 1:**
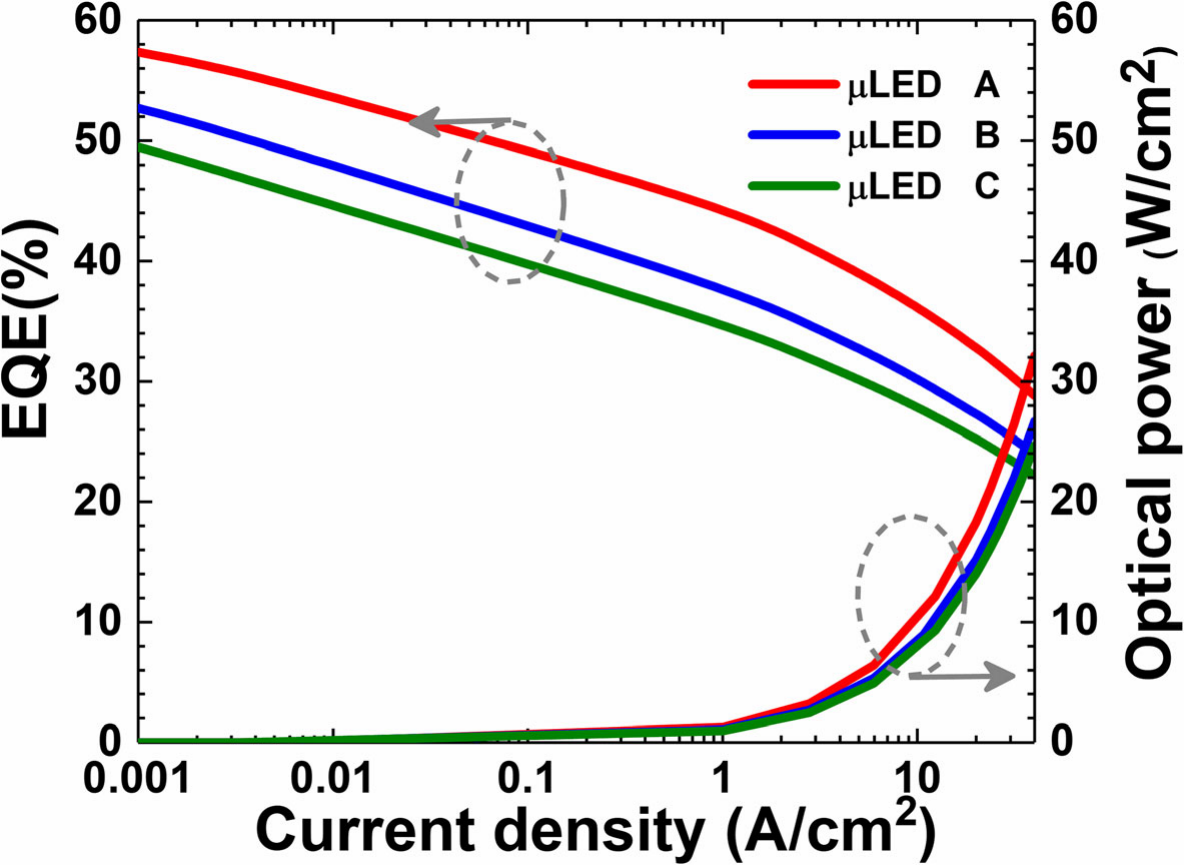
Calculated EQE and optical power density in terms of the injection current density for μLEDs A, B, and C, respectively

It is well known that a more favored hole injection can be obtained when the quantum barriers become thin [33]. However, it is not clear if thin quantum barriers help to confine current within mesas for μLEDs. For addressing the point, we here have μLEDs A, B, and C, for which the quantum barrier thicknesses, according to Table 1, are set to 6 nm, 9 nm, and 12 nm, respectively. To exclude the impact of surface recombination on the carrier distribution [18], we do not consider any traps in the mesa periphery for the investigated μLEDs. Figure 1 shows the calculated EQE and optical power in terms of the injection current density level for μLEDs A, B, and C, respectively. As shown in Fig. 1, both the EQE and the optical power increase when the quantum barrier thickness is reduced, such that the EQE values for μLEDs A, B, and C are 28.8%, 24.0%, and 22.2% at 40 A/cm^2^.

**Fig. 2:**
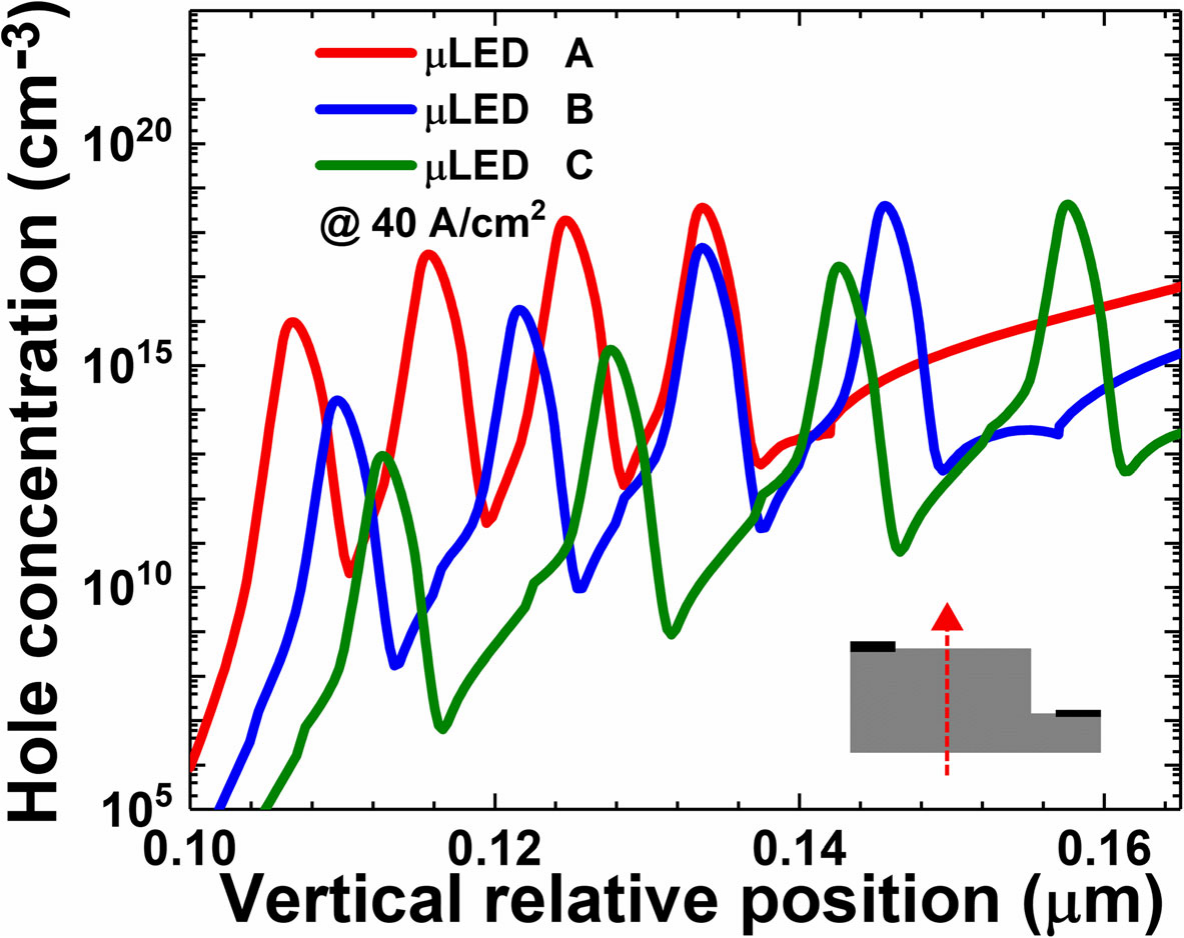
Numerically calculated hole concentration profiles in MQW regions for μLEDs A, B, and C. Data are calculated at the current density of 40 A/cm^2^. Inset figure shows the position along which the date profiles are captured

**Fig. 3:**
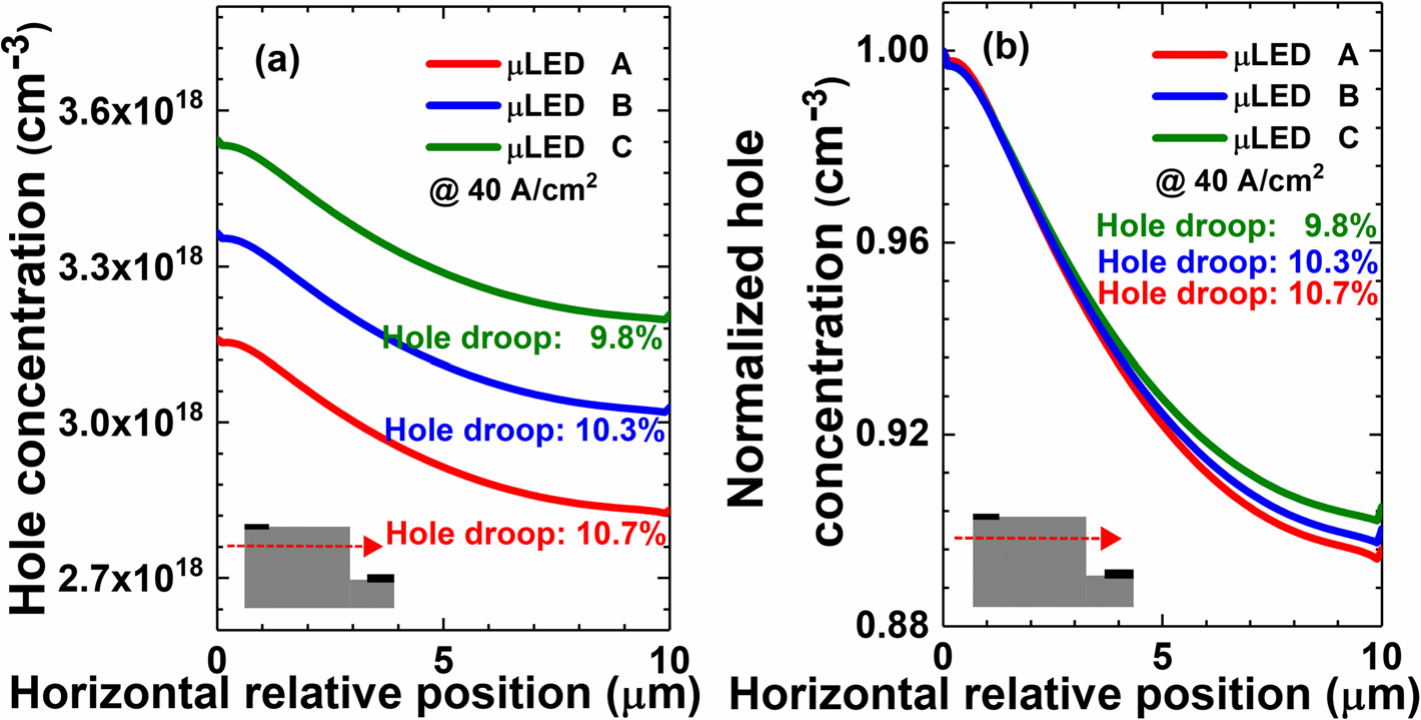
(**a**) Numerically calculated hole concentration profiles, and (**b**) normalized hole concentration profiles in the first quantum well near the p-EBL for μLEDs A, B and C, respectively. Inset figure shows the position along which the hole concentration profiles are captured. Data are calculated at the current density of 40 A/cm^2^

Figure 2 shows the hole concentration profiles in the MQW region for μLEDs A, B, and C at the current density of 40 A/cm^2^. We can see when the quantum barrier thickness is reduced, the hole concentration in the quantum wells increases. Meanwhile, the spatial uniformity for the hole distribution in the four quantum wells can also be improved. Therefore, the findings here for μLEDs are consistent with that for large-size LEDs, such that properly thin quantum barriers can promote hole transport [33]. As has been mentioned, the current can be less spreaded to the mesa edge when thin quantum barriers are adopted. We then present the lateral hole distribution in the first quantum well that is closest to the p-EBL in Fig. 3a. We find that the hole concentration decreases along with the lateral position apart from the p-electrode. We then calculate the droop level for holes, which is defined as p_left_-p_right_/p_left_. Here, p_left_ and p_right_ are denoted as the hole concentration at the left mesa edge and the right mesa edge, respectively. The droop levels are 10.7%, 10.3%, and 9.8% for μLEDs A, B, and C, respectively. For better illustration, we normalize the lateral hole concentration that is depicted in Fig. 3b. It also shows that the droop level increases as the quantum barrier becomes thin.

**Fig. 4:**
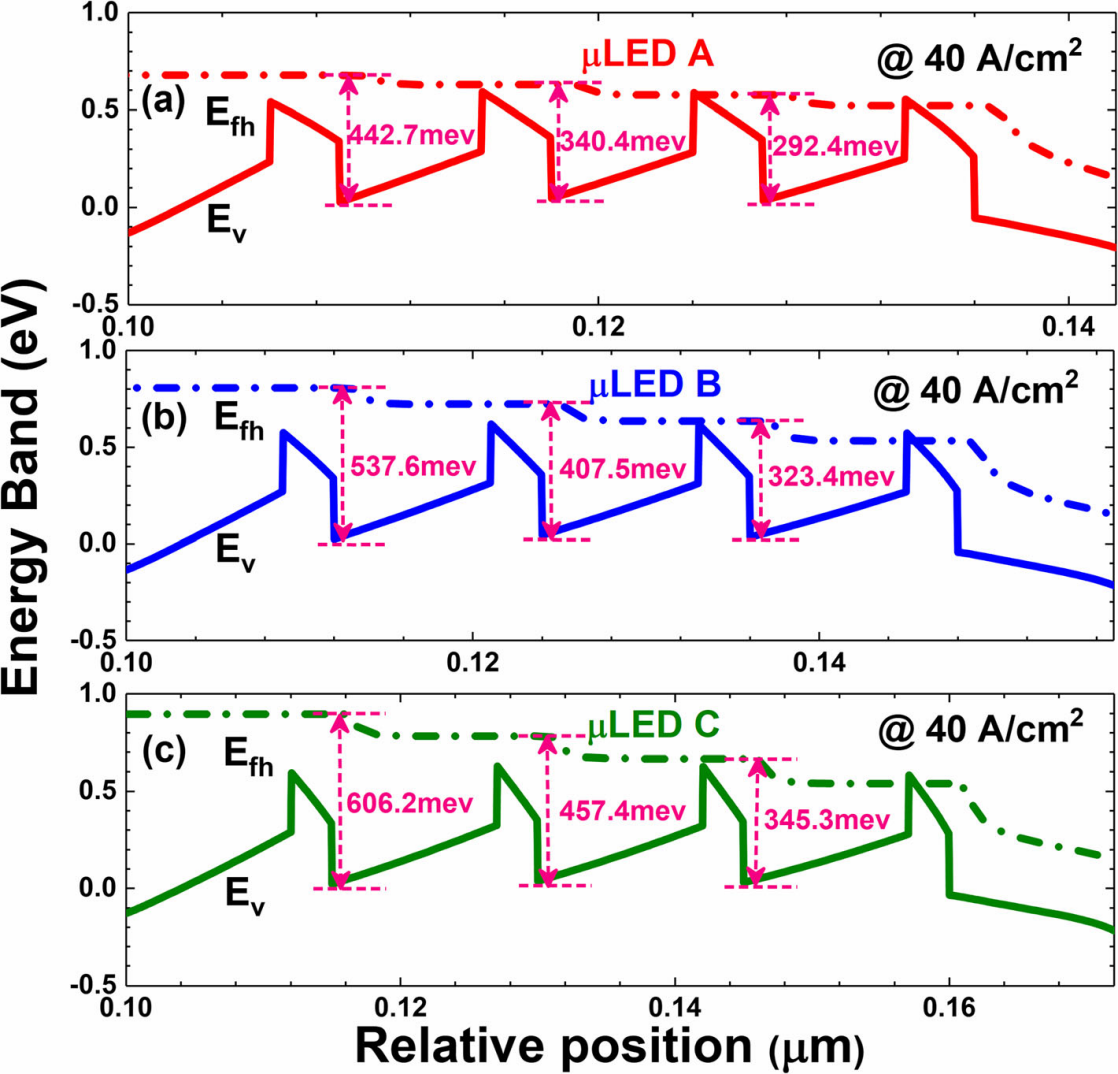
Energy band diagrams for μLEDs (**a**) A, (**b**) B, and (**c**) C. E_v_, and E_fh_ denote the valance band and quasi-Fermi level for holes, respectively. The data care calculated at the current density of 40 A/cm^2^

We then show the energy band diagrams for μLEDs A, B, and C in Fig. 4a–c. It illustrates that the valence band barrier heights for all the quantum barriers get decreased when the quantum barrier thickness reduces. The reduced valance band barrier height can better facilitate the hole transport across the MQW region, which is consistent with Fig. 2. On the other hand, when the quantum barriers are thinned, a reduced vertical resistivity will be correspondingly generated. According to the report by Che et al. [34], when the vertical resistance is reduced, the lateral current spreading can be suppressed such that the current tends to be apart from the mesa edge. This speculation is also proven when we refer to Fig. 3a and b.

**Fig. 5:**
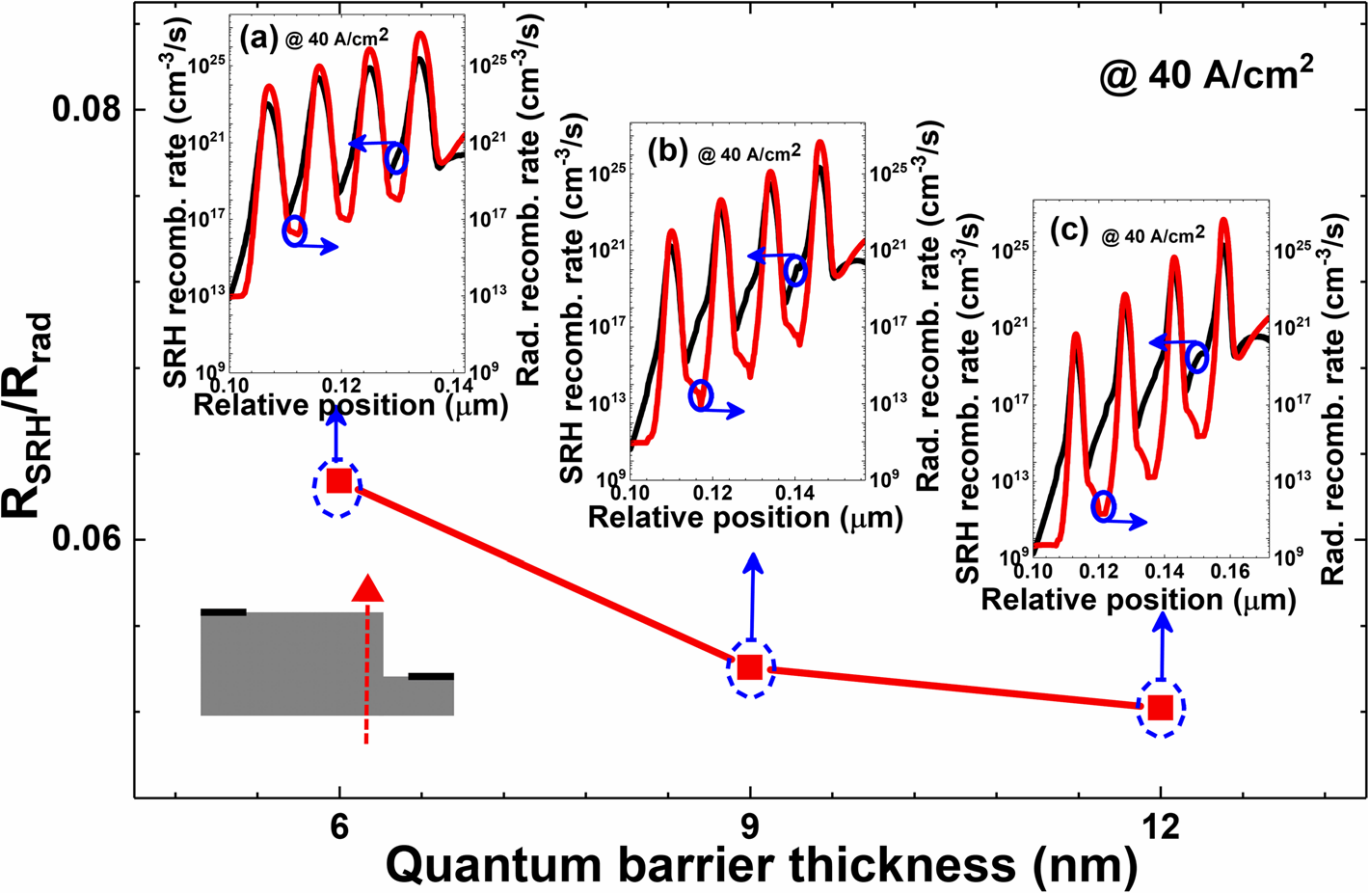
Ratios of integrated SRH recombination (SRH) rate and integrated radiative recombination rate for μLEDs A, B, and C. Insets (**a**), (**b**), and (**c**) are the profiles for SRH recombination (SRH) rate and the radiative recombination rate at the mesa edge for μLEDs A, B, and C, respectively. Data are calculated at the current density of 40 A/cm^2^

As has been mentioned above, the current spreading will be enhanced by thickening quantum barriers, which will surely affect the carrier recombination processes. We then show the ratios between the SRH recombination and the radiative recombination at the edge for the mesas (see Fig. 5). The ratio is calculated by using ${R}_{\mathrm{SRH}}/{R}_{\mathrm{rad}}={\int}_0^{{\mathrm{t}}_{\mathrm{M}\mathrm{QW}}}{R}_{\mathrm{SRH}}(x)\times \mathrm{dx}/{\int}_0^{{\mathrm{t}}_{{{}_{\mathrm{M}}}_{\mathrm{QW}}}}{R}_{\mathrm{rad}}(x)\times \mathrm{dx}$, where *R*_SRH_(x) represents the SRH recombination rate, *R*_rad_(x) denotes the radiative recombination rate, and t_MQW_ is the total thickness for MQW region. Figure 5 shows that the ratios of *R*_SRH_*/R*_rad_ both in the edge of the mesa decrease as the quantum barrier thickness increases, which means that the radiative recombination rate can be enhanced by improving the current spreading effect for ideal μLED architectures. This means that μLEDs can possess excellent current spreading because of the remarkably reduced chip size [21, 22]. Note, we have not yet considered the surface recombination for Fig. 5. Therefore, we can speculate that the much better current spreading effect for realistic μLEDs can sacrifice the carrier radiative recombination, which can be modeled by taking surface imperfections into account, and the detailed discussions will be made subsequently.

### Reduced Surface Recombination by Using MQWs with Thin Quantum Barriers

To probe the impact of the surface recombination on the hole injection for μLEDs with different quantum barrier thicknesses, we further design μLEDs I, II, and III. The structural information of the MQWs for μLEDs I, II, and III is identical to that for μLEDs A, B, and C (see Table 1), respectively except that the surface defects are considered for μLEDs I, II, and III, such that the width of the defected region for μLEDs I, II, and III is set to 0.5 μm from the etched mesa edge.

**Fig. 6:**
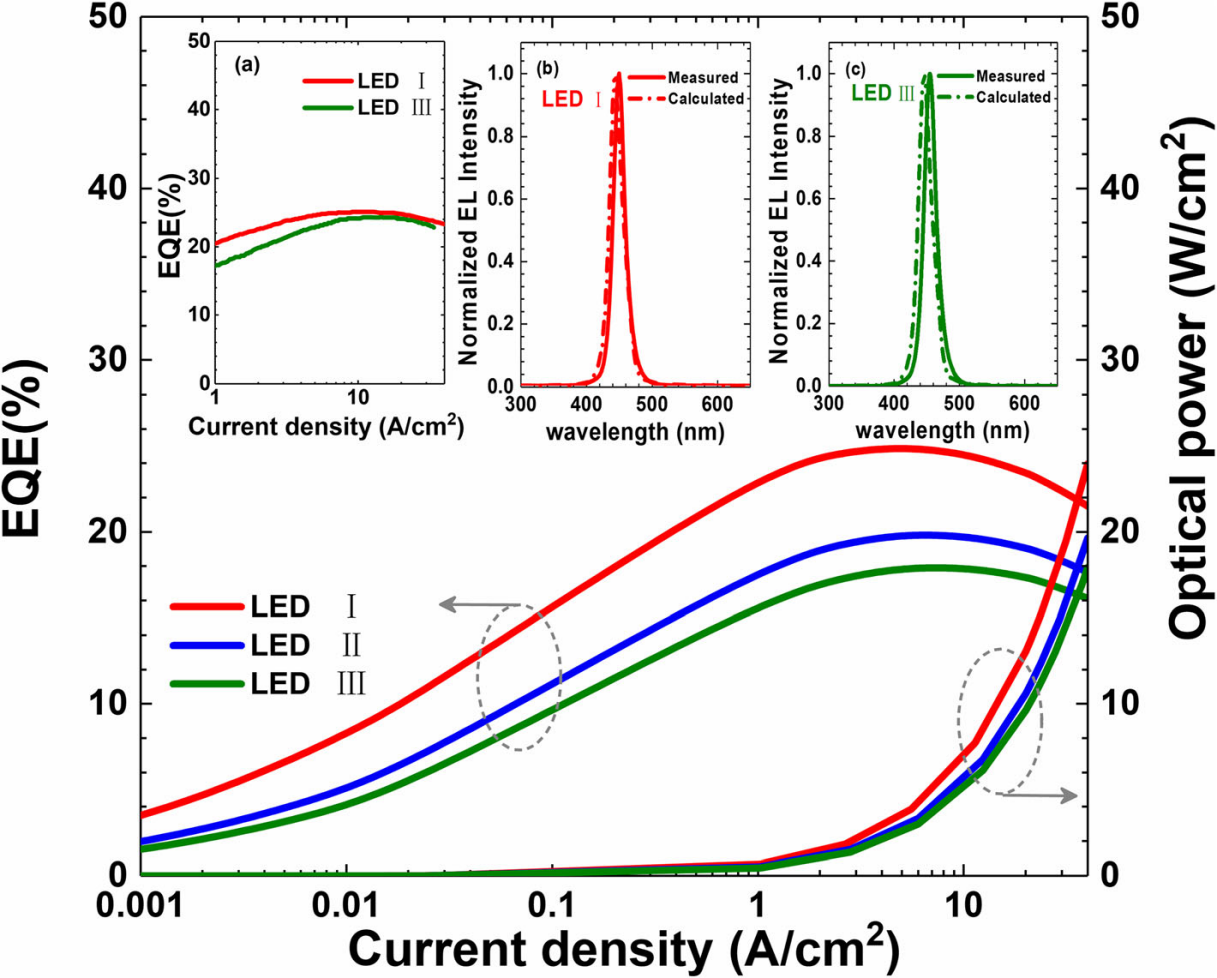
Calculated EQE and optical power density in terms of the injection current density for μLEDs I, II, and III, respectively. Inset Fig of (**a**) shows the experimentally measured EQE for μLEDs I and III, respectively. Inset figures of (**b**) and (**c**) present the measured and numerically calculated EL spectra for μLEDs I, and III. Data for inset Figs (**b**) and (**c**) are collected at the current density of 40 A∕cm^2^

The numerically computed EQE and optical power as a function of the current density are demonstrated in Fig. 6. Figure 6 shows that when surface nonradiative recombination is considered, the optical intensity can be significantly decreased. Therefore, this further confirms that the surface nonradiative recombination cannot be ignored for μLEDs [10, 17, 18]. In the meantime, agreeing well with the observations in Fig. 1, the EQE and the optical power also get enhanced when the quantum barrier thickness decreases, e.g., μLED I with the thinnest quantum barrier has the largest EQE and optical power. The experimentally measured EQE for μLEDs I and III are shown in inset Fig. 6a, which shows the same trend as the numerical calculation results. In addition, we measure and show the normalized electroluminescence (EL) spectra for μLEDs I and III in Fig. 6b and c, respectively. The peak emission wavelength for all the tested μLEDs is ~450 nm. The measured EL can be reproduced by our models. This indicates that the physical parameters we have utilized are set correctly, e.g., the polarization level and the InN composition in the MQWs that determine the emission wavelength have been properly set.

**Fig. 7:**
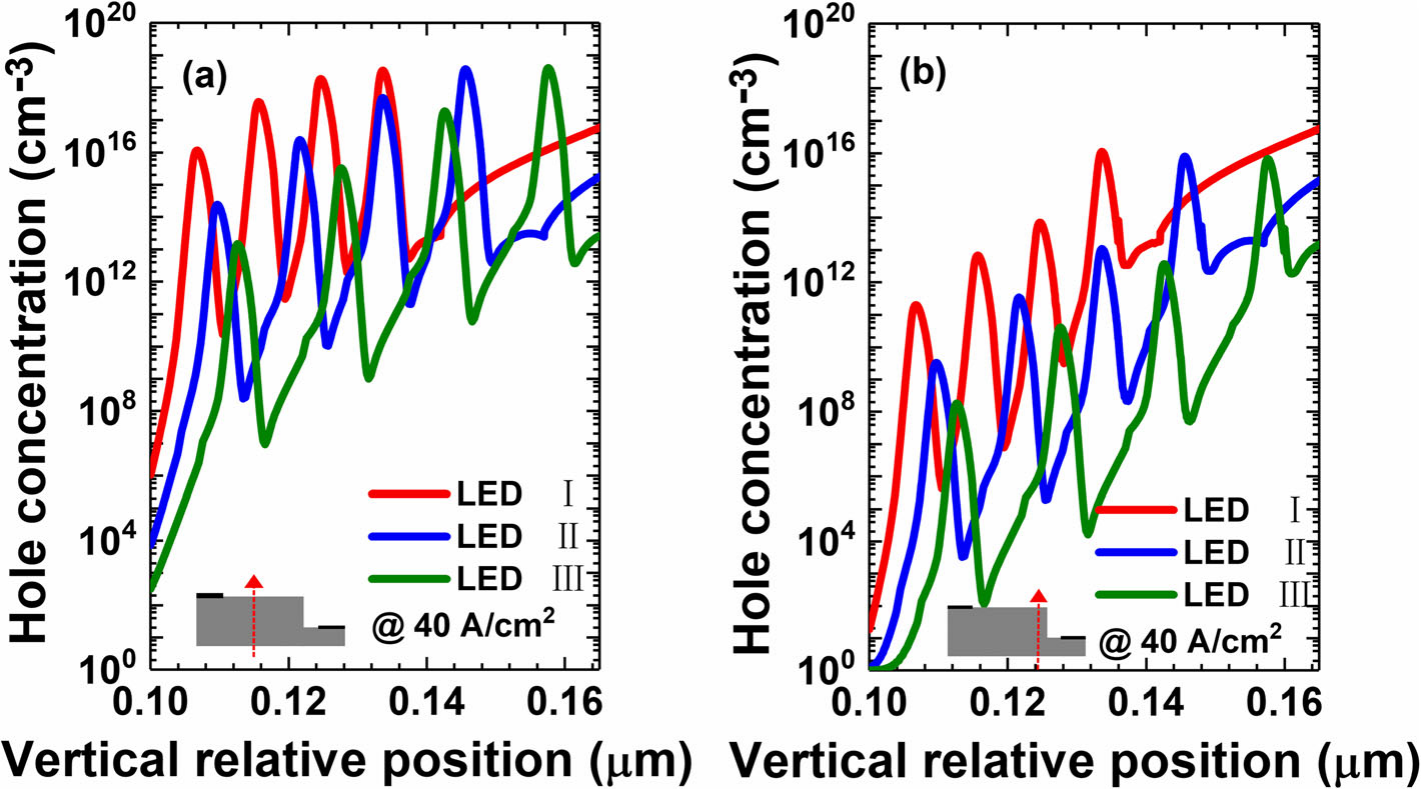
Numerically calculated hole concentration profiles in the MQW region (**a**) in the center, (**b**) at the edge of the mesas for μLEDs I, II, and III, respectively. Data are calculated at the current density of 40 A/cm^2^. Inset figures show the position along which the hole concentration profiles are captured

In order to reveal the effect of the sidewall defects on the hole injection efficiency for μLEDs I, II, and III, the hole concentrations are shown in Fig. 7. Note, the hole concentration in Fig. 7a is probed in the middle region for the devices [as indicated by the red arrow in the inset of Fig. 7a]. Figure 7b shows the hole concentration in the defected region for the devices [as indicated by the red arrow in the inset of Fig. 7b]. As Fig. 7a and b illustrate, for both the non-defected region and the sidewall region, the reduced thickness for quantum barriers favors the hole transport across the MQWs. The results here are consistent with Fig. 2. Further comparison between Fig. 7a and b shows that hole injection efficiency at the defected sidewall regions is obviously lower than that in the non-defected region. The observations here agree well with Kou et al. [18], which further manifests that it is essentially required to make current less spread to the defected sidewalls by properly reducing the quantum barrier thickness (see Fig. 3a and b).

**Fig. 8:**
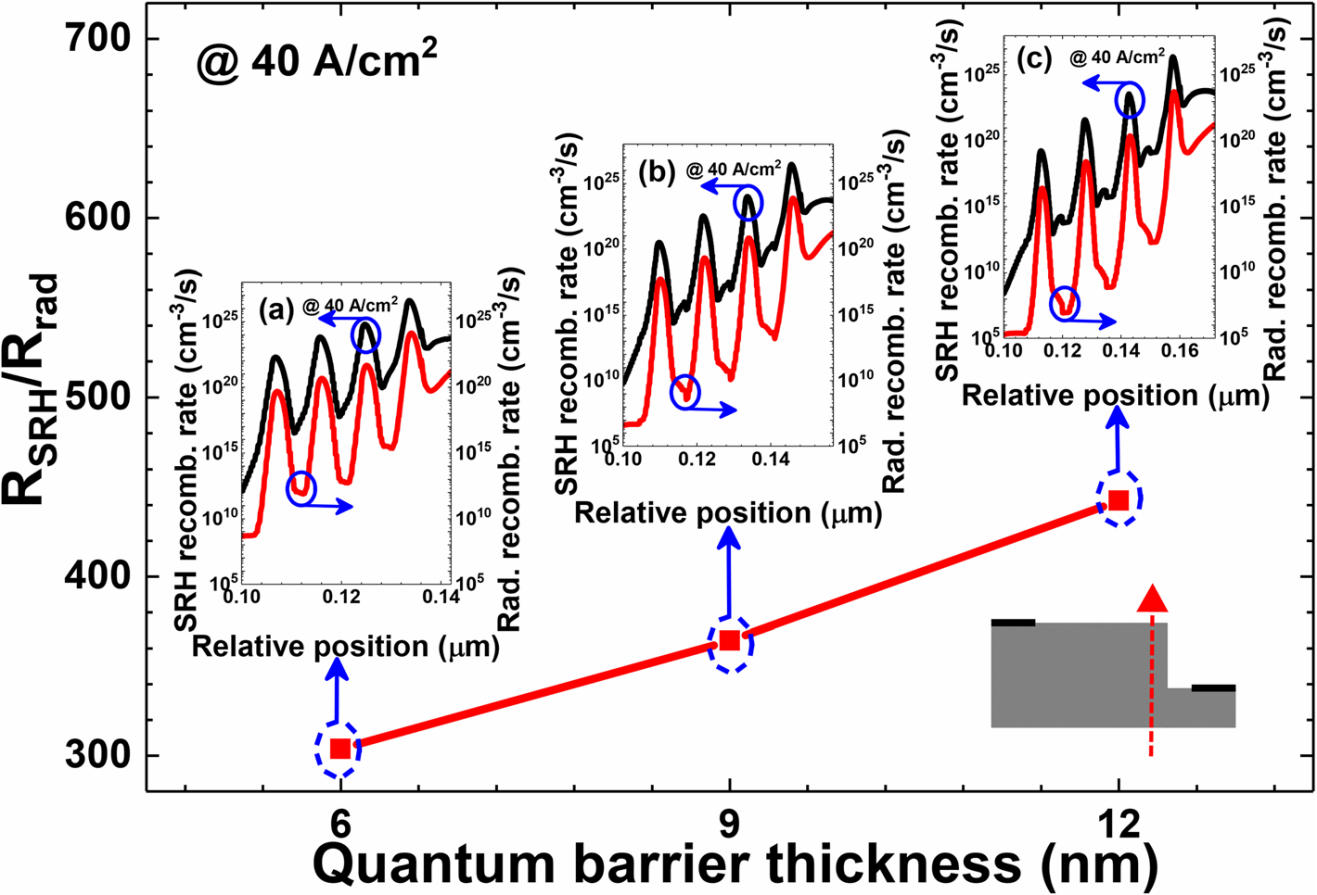
Ratios of the integrated SRH recombination (SRH) rate and the integrated radiative recombination rate for μLEDs I, II, and III. Inset figures (**a**), (**b**), and (**c**) are the profiles for SRH recombination (SRH) rate and the radiative recombination rate at the mesa edge for μLEDs I, II, and III, respectively. Data are calculated at the current density of 40 A/cm^2^

We then repeat our analysis as we have done in Fig. 5, the values for which are now demonstrated in Fig. 8. We can see that the ratio for *R*_SRH_*/R*_rad_ at the mesa edge increases when the quantum barrier is thickened, which is uniquely ascribed to the significantly enhanced surface nonradiative recombination rate. As we have proposed, thick quantum barriers allow the current to arrive at mesa edges and trigger the surface nonradiative recombination. As a result, inset Fig. a–c also shows that the surface nonradiative recombination becomes extremely strong at mesa edges. The nonradiative recombination rate at sidewalls even overwhelms the radiative recombination rate.

## Conclusions

In summary, we have numerically investigated and demonstrated the impact of different quantum barrier thicknesses on the hole injection and the current spreading for InGaN-based μLEDs. The results indicate that by thinning the quantum barrier thickness, a better current confinement within the mesa region can be enabled. Correspondingly, the current spreading can be well managed to be apart from mesa edges, which then suppresses surface nonradiative recombination. Both numerically and experimentally, we observe the improved external quantum efficiency for InGaN-based μLEDs with properly thin quantum barriers. We believe that the proposed approach is promising for removing the bottleneck that limits the development of high-performance μLEDs. Moreover, the device physics that is presented in this work will increase the understanding of InGaN-based μLEDs.

## Data Availability

The data and the analysis in the current work are available from the corresponding authors on reasonable request.

## Abbreviations

μLEDs: Micro-light-emitting diodes
EQE: External quantum efficiency
LEDs: Light-emitting diodes
InGaN: Indium gallium nitride
GaN: Gallium nitride
VLC: Visible light communication
IQE: Internal quantum efficiency
PECVD: Plasma-enhanced chemical vapor deposition
ALD: Atomic layer deposition
MQWs: Multiple quantum wells
MOCVD: Metal-organic chemical vapor deposition
p-EBL: p-Type electron blocking layer
APSYS: Advanced Physical Models of Semiconductor Devices
SRH: Shockley-Read-Hall
